# mPGES-1-Mediated Production of PGE_2_ and EP4 Receptor Sensing Regulate T Cell Colonic Inflammation

**DOI:** 10.3389/fimmu.2018.02954

**Published:** 2018-12-14

**Authors:** Damian Maseda, Amrita Banerjee, Elizabeth M. Johnson, Mary Kay Washington, Hyeyon Kim, Ken S. Lau, Leslie J. Crofford

**Affiliations:** ^1^Department of Pathology, Microbiology, and Immunology, Vanderbilt University Medical Center, Nashville, TN, United States; ^2^Division of Rheumatology and Immunology, Department of Medicine, Vanderbilt University Medical Center, Nashville, TN, United States; ^3^Department of Cell and Developmental Biology, Epithelial Biology Center, Vanderbilt University School of Medicine, Nashville, TN, United States

**Keywords:** IBD–inflammatory bowel diseases, T cell, PGE2, colitis, Th17 & Tregs cells, inflammation immunomodulation, Th17 activation, Treg = regulatory T cell

## Abstract

PGE_2_ is a lipid mediator of the initiation and resolution phases of inflammation, as well as a regulator of immune system responses to inflammatory events. PGE_2_ is produced and sensed by T cells, and autocrine or paracrine PGE_2_ can affect T cell phenotype and function. In this study, we use a T cell-dependent model of colitis to evaluate the role of PGE_2_ on pathological outcome and T-cell phenotypes. CD4^+^ T effector cells either deficient in mPGES-1 or the PGE_2_ receptor EP4 are less colitogenic. Absence of T cell autocrine mPGES1-dependent PGE_2_ reduces colitogenicity in association with an increase in CD4^+^RORγt^+^ cells in the lamina propria. In contrast, recipient mice deficient in mPGES-1 exhibit more severe colitis that corresponds with a reduced capacity to generate FoxP3^+^ T cells, especially in mesenteric lymph nodes. Thus, our research defines how mPGES-1-driven production of PGE_2_ by different cell types in distinct intestinal locations impacts T cell function during colitis. We conclude that PGE_2_ has profound effects on T cell phenotype that are dependent on the microenvironment.

## Introduction

Prostaglandin E_2_ (PGE_2_) is an eicosanoid that modulates diverse physiologic and pathologic functions. To avoid undesired effects associated with dysregulated inflammation, PGE_2_ tissue concentrations are tightly regulated by expression of constitutive (COX1) and inducible (COX2, mPGES1) biosynthetic enzymes, as well as degradative enzymes (15-PGDH) (1, 2) and the transporters multiple drug resistance-associated protein 4 (MRP4) and prostaglandin transporter (PGT). The COX1 and COX2 enzymes are responsible for the catabolism of arachidonic acid into PGH_2_, the precursor of PGI_2_, PGD_2_, PGF_2a_, TxA_2_, and PGE_2_. COX1 and 2 expression differs strongly among different tissues, and distinct inflammatory triggers markedly upregulate the inducible isoform, COX2 (2, 3). mPGES1, also an inducible enzyme often co-regulated with COX-2, acts downstream of COX enzymes to specifically generate PGE_2_, and does not directly generate other prostaglandins that are derived from the shared PGH_2_ intermediate metabolite. Part of the variety of effects that can be triggered by PGE_2_ are due to its 4 known receptors (EP1-4), which display different PGE_2_ binding affinities and a range of tissue and cell-specific expression patterns (2, 4). PGE_2_ signaling in T cells is almost exclusively transduced through the EP2 and EP4 receptors (5, 6).

Immune responses can be highly sensitive to PGE_2_, as it acts in a pleiotropic manner affecting many cells of the immune system. With respect to the innate immune system, PGE_2_ promotes neutrophil, macrophage, and mast cell activation and migration into inflamed sites, and can also influence dendritic cells (DC) with both pro- and anti-inflammatory results (7–9). Cells belonging to the adaptive immune system can also be influenced by PGE_2_, and T cells display an array of diverse responses to PGE_2_ that include effects on T cell lineage commitment and cytokine secretion (10–13). In general, PGE_2_ can differentially expand Th1 and Th17 cells via expression of the PGE_2_ receptors EP2 and EP4 when IL1β and IL-23 are part of the inflammatory response. However, some of the reported effects are contradictory, as PGE_2_ can either increase IL-17 and reduce IFNγ production in human memory T cells (14) and inhibit Th1 responses (15), or enhance antigen-specific Th1 function and expansion (11). We recently found that production and sensing of PGE_2_ in CD4^+^ T cells controls antigen-specific regulatory T cell (Treg) and Th17 expansion, and that PGE_2_ can re-direct T cells undergoing Th17 polarization to inhibit IL-17A production in lieu of IFNγ (13).

In the gastrointestinal tract, PGE_2_ is implicated in the modulation of autoimmune and inflammatory diseases as well as in the control of carcinogenesis (16–18). Expression of COX-2 is greatly increased during active phases in patients with ulcerative colitis (19), and non-steroidal anti-inflammatory drugs (NSAIDs) are associated with significant gastrointestinal adverse events and exacerbate symptoms of inflammatory bowel disease (IBD) (20). COX-2 is partially responsible for intestinal damage in the DSS colitis model in mice, with presence of COX-2 being required specifically in myeloid and endothelial cells but not epithelial cells (21) to increase intestinal pathology. However, although PGE_2_ is generally regarded as a pro-inflammatory molecule, it is also critical for the resolution of inflammation and restoration of tissue homeostasis (2, 22).

It is unclear how PGE_2_ affects pathogenic T cell responses during colitis, and what the relative contribution of T cell intrinsic or extrinsic sources might be to clinical disease. Given the effects of PGE_2_ on T cells, PGE_2_ could constitute a critical regulator of Th17 cells in the colon, which can either enhance protective responses from gut pathogens and epithelial regeneration, but also potentially serving to mediate IBD (23–25). In this study we evaluated the impact of mPGES1-driven PGE_2_ and PGE_2_ sensing through EP4 in a T-cell driven colitis model. We found that signaling through EP4 on CD4^+^ cells strongly controlled colitogenicity and colonic T cell expansion. Moreover, our studies comparing the origin of PGE_2_ production in T cells compared with non-lymphoid cells demonstrate that T cell autocrine mPGES1-mediated PGE_2_ contributes to colitogenesis by reducing a protective CD4^+^RORγt^+^ cell response, while paracrine mPGES-1-driven PGE_2_ inhibits colon inflammation by favoring the expansion of FoxP3^+^ Treg cells. We conclude that site-specific PGE_2_ production and PGE_2_ sensing by CD4^+^ T cells play important roles in intestinal inflammation.

## Materials and Methods

### Mice and Colitis Induction

WT and mPGES1^−/−^ mice in a BL/6 background were bred in house and maintained under SPF conditions in the same MCN II facility and room at Vanderbilt University. mPGES-1 mice were originally obtained from Pfizer and their generation has been previously described (26). EP4^fl/fl^ transgenic mice were a kind gift of Dr. Richard Breyer (27), and they were crossed with either C57BL/6 or C57BL/6 CD4^Cre^ mice obtained from Jackson Laboratories. Rag1^−/−^ mice were obtained from Jackson Laboratories and crossed with the mPGES1^−/−^ line to obtain double knock-outs. All mice were bred in a specific pathogen-free barrier facility and used at 8–14 weeks of age. The Vanderbilt University Animal Care and Use Committee approved all studies performed for the preparation of this manuscript.

Colitis was induced by adaptive transfer of 1 × 10^6^ purified (>99% purity) CD4^+^CD25^−^CD45RB^hi^ cells i.p., and in the indicated cases co-injection of 0.5 × 10^6^ CD4^+^CD25^−^ cells was performed to study Treg function. Spleen and lymph nodes suspensions were used first to purify untouched CD4^+^ cells using magnetic bead cell separation with a StemCell Kit and these cells were stained with anti-CD4, anti-CD25, and anti-CD45RB for further flow sorting using a FACS Diva flow cytometer (Becton-Dickinson) with purities over 95% of the indicated populations. Mice that received adoptive transfers of different cell genotypes were always cohoused in the same cages to avoid differences due to microbiota composition divergence during colitis development.

### Cell Preparation and Flow Cytometry

Single cell suspensions were prepared from spleen, colon LP, and mesenteric lymph nodes, and stained on ice using predetermined optimal concentrations of each antibody (Ab) for 20–30 min, washed, and fixed using 1.5% PFA or eBioscience FoxP3 fixation reagent. Colon lamina propria was obtained as previously described (28). Cells with the light scatter properties of singlet lymphocytes were analyzed by multicolor immunofluorescence staining and a BD FACS Fortessa II flow cytometer (Becton Dickinson, San Jose, CA). Fcγ receptors blockade was performed (2.4G2; BD PharMingen) prior to surface staining of cell surface markers. The anti-mouse mAbs used in this study included CD4-PE.Cy7/FITC (GK1.5), CD45.1-AF700/Pacific Blue (A20), CD45RB-AF647 (C363-16A) from BioLegend, RORγt-PE (Q31-378) from BD PharMingen, FoxP3-APC (FJK-16s) from eBioscience, and CD45.2-PE (104) from Tonbo. The LIVE/DEAD® fixable cell death stain kit (Invitrogen) was used to remove dead cells from all analysis and avoid background staining noise of dead cells. All flow cytometry analysis and data display were performed using FlowJo software.

### Tissue Culture and PGE_2_ Measure

For all *in vitro* experiments IMDM medium was supplemented with 10% FCS, Pen/Strep at 50 UI/ml and 50 μg/ml respectively, and 2-beta-ME at 10 μM. Colon explant cultures were performed in 48-well round-bottom plates and supernatants were collected 12 h after initiation, spin down at >12.000 g in Eppendorf tubes, and clear supernatants used for further analysis. NS-398 was purchased from Cayman Chemicals, and stored aliquots were freshly reconstituted before every use.

The PGE_2_ ELISA Kit from Cayman chemical was used to evaluate PGE_2_ supernatant concentrations.

### Histology and Pathological Scoring

Colon Swiss rolls were generated from mice undergoing colitis at the indicated time-points. Fresh colon tissue was washed with cold PBS, cut longitudinally to prepare Swiss rolls and fixed in 10% Formaldehyde for 3 days before transfer to 70% Ethanol. Paraffin blocks were generated with these fixed samples and 10 μm sections placed in slides for further H&E processing. Pathological severity and features were evaluated according to the following scoring system: Lamina Propria Infiltrate (LPI, 0–3), Neutrophilic Infiltrate (NI, 0–2), Goblet Cell Loss (GCL, 0–3), Abnormal Crypts (Ab.Cr., 0–3), Crypt Abscesses (Cr. Ab., 0–1), Erosion and Ulcers (Er.+Ulc, 0–2), and Depth of Inflammation (DOI, 0–3). Scale bars on the images correspond to 100 μM length. For detection of COX2 and mPGES-1 in colon tissue, we used rabbit polyclonal anti-mouse COX2 ab52237 and anti-mouse mPGES-1 ab62050 from Abcam following manufacturer's instructions.

### Microscopy Analysis, Immunofluorescence and Signal Quantification

Paraffin-embedded colonic tissues were sectioned (5 μm) prior to deparaffinization, rehydration, and antigen retrieval using a citrate buffer (pH 6.0) for 20 min in a pressure cooker at 105°C, followed by a 20 min cool down at room temperature (RT). Endogenous background signal was quenched by incubating tissue slides in 3% hydrogen peroxide for 10 min at RT. Tissues were blocked in 3% BSA/10% donkey serum for 1 h before primary Ab staining. Antibodies used for immunofluorescence were: rat anti-FoxP3-APC (1:100, eBioScience FJK-16a), mouse anti-RORγt-PE (1:100, BD Q31-378), goat anti-CD3ε (1:100, Santa Cruz M-20), rabbit anti-pSTAT3 (Tyr705) (1:100, Cell Signaling D3A7), and AF-647-conjugated secondary antibodies (Life Technologies). Sequential staining and fluorescent dye inactivation was performed as previously described (29, 30). Immunofluorescent imaging was performed using an Olympus X81 inverted microscope with an UPlanSAPO UIS2 (20X/0.75NA) objective lens and filter sets specific for DAPI, GFP, CY3, CY5, and Cy7. Images were acquired at 20X magnification and image exposure for each Ab stain was set manually (< 800 ms). Initial surveying of the tissue was performed at 2X magnification on the DAPI channel to establish 10–15 regions per Swiss roll for subsequent analysis. Primary Ab staining was performed overnight at RT and secondary Ab staining for 1 h at RT before slide imaging. Complete inactivation of fluorochromes was performed as described previously (29). Final image processing and layering was performed using ImageJ.

### Microscopy Imaging Processing, Single-Cell Quantification, and Data Analysis

Images acquired for each stain round were processed as previously described (29). For each stain round, DAPI images were aligned to those from the first round using rigid transformation. Autofluorescence removal and correction was performed by background subtraction of registered images. Autofluorescence removed images for each stain were used for single-cell segmentation using Mathworks MATLAB software. Expression values of transcription factors were quantified by median intensity levels within a given mask-generated nuclear segmentation using combination of all nuclear markers available. CD3ε^+^ cell numbers were estimated by the total area of coverage per field of the CD3ε^+^ mask, divided by the average area of a single CD3ε^+^ cell. This estimate was verified manually by counting CD3ε^+^ cells in selected fields of view and comparing to estimated values. All analyses were performed in a blind fashion without phenotype identifiers. Cytobank was utilized to analyze single-cell intensity values and quantify cell populations.

## Results

### Production of PGE_2_ in the Colon Is Regulated by mPGES-1 With the Contribution of T and B Cells

**Figure 1:**
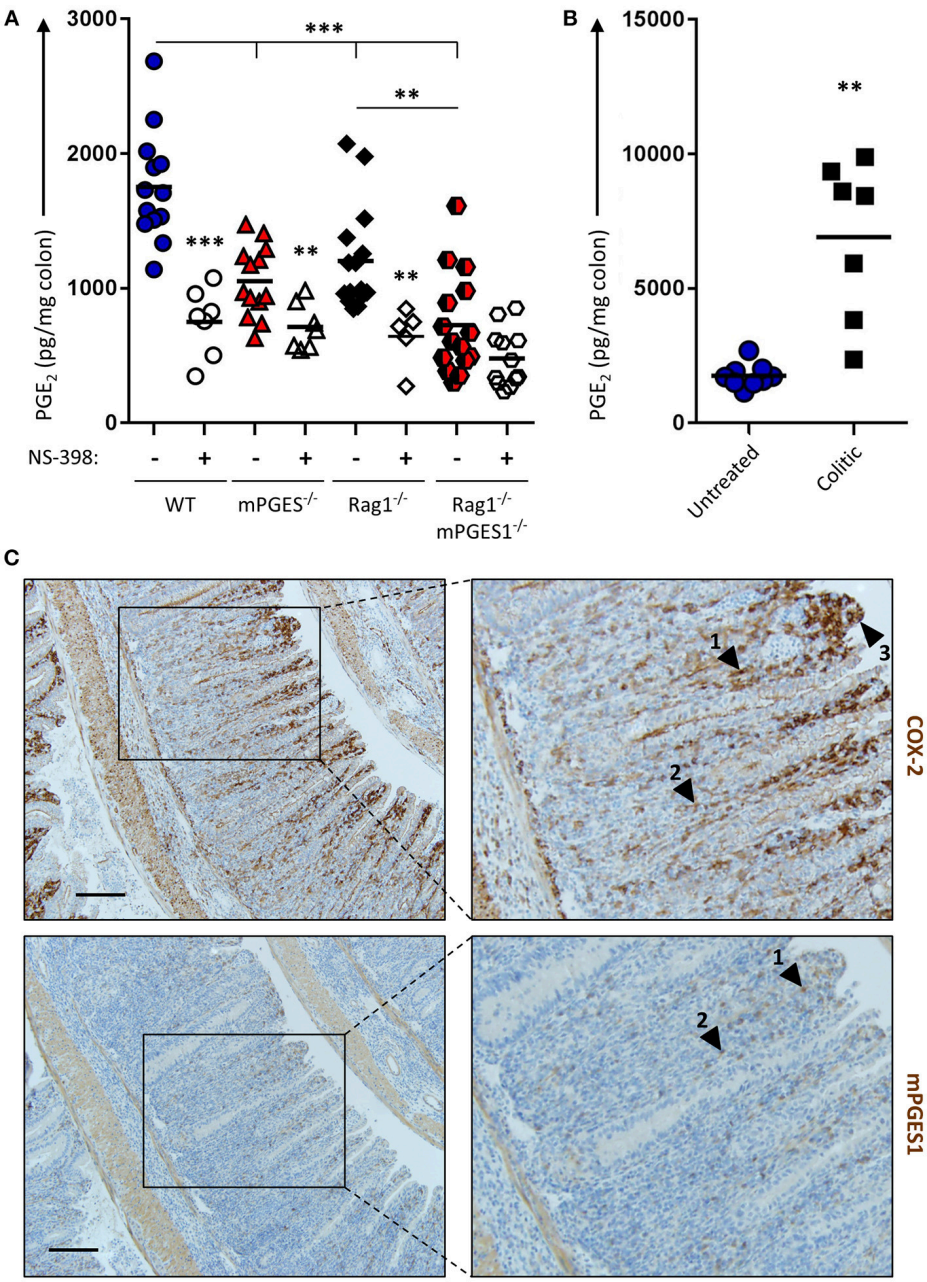
Basal production of PGE_2_ in the colon is regulated by COX-2 and mPGES-1 with significant contribution from cells of the adaptive immune system. **(A)** Colon explants from WT (blue), mPGES-1^−/−^ (red), Rag1^−/−^ (black) or Rag1^−/−^ × mPGES-1^−/−^ (red/black) mice were cultured for 12 h and their supernatants were analyzed for basal PGE_2_ production. The specific COX2 inhibitor NS-398 was added to evaluate the differential contribution of COX2 and COX1 to the PGH_2_ pool prior to PGE_2_ biosynthesis. **(B)** PGE_2_ production in colon explants from untreated WT mice (“Untreated,” blue circles) or Rag1^−/−^ mice undergoing WT Teff cell-driven colitis at week 10 post-transfer (“Colitic,” black squares). ** indicates a *P* value < 0.05 using a 2-tailed heteroscedastic Student's *T*-test. **(C)** Immunohistochemistry of COX2 (upper row) and mPGES-1 (lower row) in the colon of mice that transferred with WT Teff cells (CD4^+^CD25^−^CD45RB^hi^). Scale bar is 100 μm long. Arrowheads 1 and 2 point to localization within the LP, while arrowhead 3 indicates localization in the brush border. ** Significant at *P* < 0.05 and *** at *P* < 0.01 in a one-way ANOVA with Welch's correction.

The intestines are well-known to generate PGE_2_, and inhibition of COX enzymes reduces PGE_2_. However, it has not been investigated to which extent different components of the intestines contribute to this PGE2 pool. To address this and to evaluate how baseline production of PGE_2_ is influenced by both biosynthetic enzymes and by the presence of lymphocytes, we cultured colon explants of untreated Rag1^−/−^, mPGES-1^−/−^, or Rag1^−/−^ × mPGES-1^−/−^ double knockout mice overnight and their supernatants were assessed for PGE_2_ concentration. As expected, colons of WT animals produced the highest amounts of PGE_2_. Absence of mPGES-1 significantly impaired PGE_2_ production, but so did too the lack of adaptive immune cells in the Rag1^−/−^ group (Figure 1A). Interestingly, lack of both the mPGES-1 enzyme and lymphocytes demonstrated an additive effect, with Rag1^−/−^ × mPGES-1^−/−^ double knockouts exhibiting the lowest levels of PGE_2_. Specific inhibition of COX-2 activity using NS-398 in these explant cultures resulted in a reduction of nearly half of the secreted PGE_2_ under most conditions_._ However, a significant decrease in PGE_2_ was not observed following COX-2 inhibition in colons of Rag1^−/−^ × mPGES-1^−/−^ double knockout mice likely due to the already low basal levels produced.

We hypothesized that colonic inflammation would increase the PGE_2_ production capacity in the colon. To test this, we assessed PGE_2_ concentration in colon explants of WT untreated mice or Rag1^−/−^ mice undergoing colitis induced by adoptive transfer of effector CD4^+^ cells. Colons of mice undergoing colitis at week 10 post-transfer depicted a 5-fold increase of PGE_2_ compared to healthy untreated colons (Figure 1B).

Previous studies have shown that the COX-2 and mPGES-1 enzymes are especially abundant in monocyte/macrophage cells, and COX-2 expression is restricted in the absence of inflammation to the kidney, gastrointestinal tract, brain, and thymus (31). To better understand the increase of PGE_2_ observed during colitis, we collected, fixed and stained colitic colons to detect both enzymes. COX-2 expression was maximal in the lamina propria (LP) at the apical side of hyperplastic villi, and mPGES-1 expression was found to mimic that pattern to some extent (Figure 1C, arrowheads #1-2), although strong COX-2, but not mPGES-1 expression, was observed in the brush border of the villi (arrowhead #3).

### CD4^+^ Effector T Cells Lacking mPGES-1 Have Impaired Colitogenic Potential

**Figure 2:**
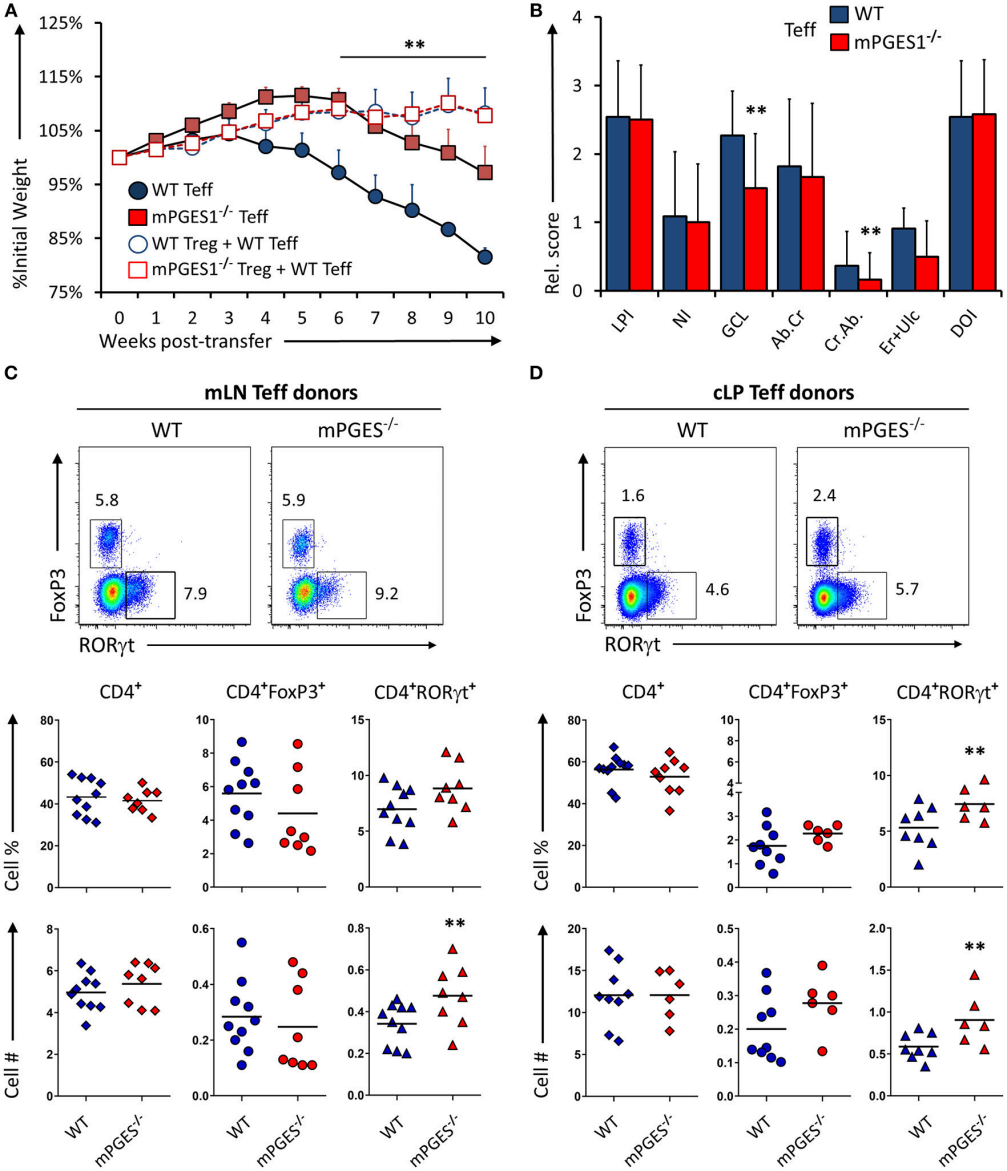
mPGES-1-deficiency in T effector cells protects against colitis. **(A)** Weight loss in Rag1^−/−^ mice that received transfer of 1 × 10^6^ CD4^+^CD25^−^CD45RB^hi^ T cells (Teff) from WT or mPGES-1^−/−^ donors. The dashed lines correspond to mice that received WT Teff and WT or mPGES-1^−/−^ CD4^+^CD25^+^ (Treg) co-transfers. **(B)** Colon pathology scores from cohorts receiving transfers of WT or mPGES-1^−/−^ Teff cells. Flow cytometry analysis of the **(C)** mesenteric lymph nodes (mLN) and **(D)** colon lamina propria (cLP) CD4^+^ populations at the end of the experiment (week 10), with representative dot plots indicating intracellular expression of RORγt and FoxP3 and graphs below indicating summarized results from 4 experiments. ** indicates a significant difference with *P* < 0.05 using a two-tailed heteroscedastic Student's *T*-test between the WT and mPGES-1^−/−^ Teff donor groups.

We have recently demonstrated that T cell autocrine mPGES-1 expression in CD4^+^ cells contributes to the cytokine profile that CD4^+^ cells can acquire during antigen-specific stimulation. This T cell intrinsic effect is synergic with the ability to secrete PGE_2_ by interacting APC cells, and it impacts especially IL-17A and IFNγ production by CD4^+^ cells (13). We asked ourselves if this T cell intrinsic mPGES-1-driven PGE_2_ effect was also contributing to the phenotype and pathogenic potential of T cells in a colitis model. Rag1^−/−^ mice received an adoptive transfer of CD4^+^CD25^−^CD45B^hi^ cells (Teff) from WT or mPGES-1^−/−^ donor mice, and were monitored and evaluated for colitis progression for 10 subsequent weeks after transfer. Mice that received mPGES-1^−/−^ Teff CD4^+^ cells demonstrated a less severe weight loss than mice receiving WT Teff CD4^+^ cells at the latter phases of disease (Figure 2A). Co-transfer of WT Treg cells with WT Teff cells resulted in suppression of colitis as expected (Figure 2A, empty circles), and mPGES-1^−/−^ Tregs also displayed full protective function indistinguishable from WT Tregs in terms of weight loss (empty squares). Analysis of the pathological characteristics in the colons of both groups transferred with Teff cells revealed that mice that received mPGES-1^−/−^ Teff CD4^+^ cells lost less goblet cells and developed less crypt abscesses (Figure 2B). To investigate if the attenuation of colitis was due to an altered phenotype of the transferred T cells, we analyzed by flow cytometry the expression of FoxP3 and RORγt in CD4^+^ cells in the mesenteric lymph nodes (mLN) and colonic lamina propria (cLP) of the mice belonging to the same colitis cohorts described above. mPGES-1^−/−^ Teff CD4^+^ cells were able to generate moderately but significantly increased numbers of CD4^+^RORγt^+^ T cells in the mLN and the cLP (Figures 2C,D) with the percent being increased only in the cLP. CD4^+^FoxP3^+^ cells and total CD4^+^ cells were not altered.

**Figure 3:**
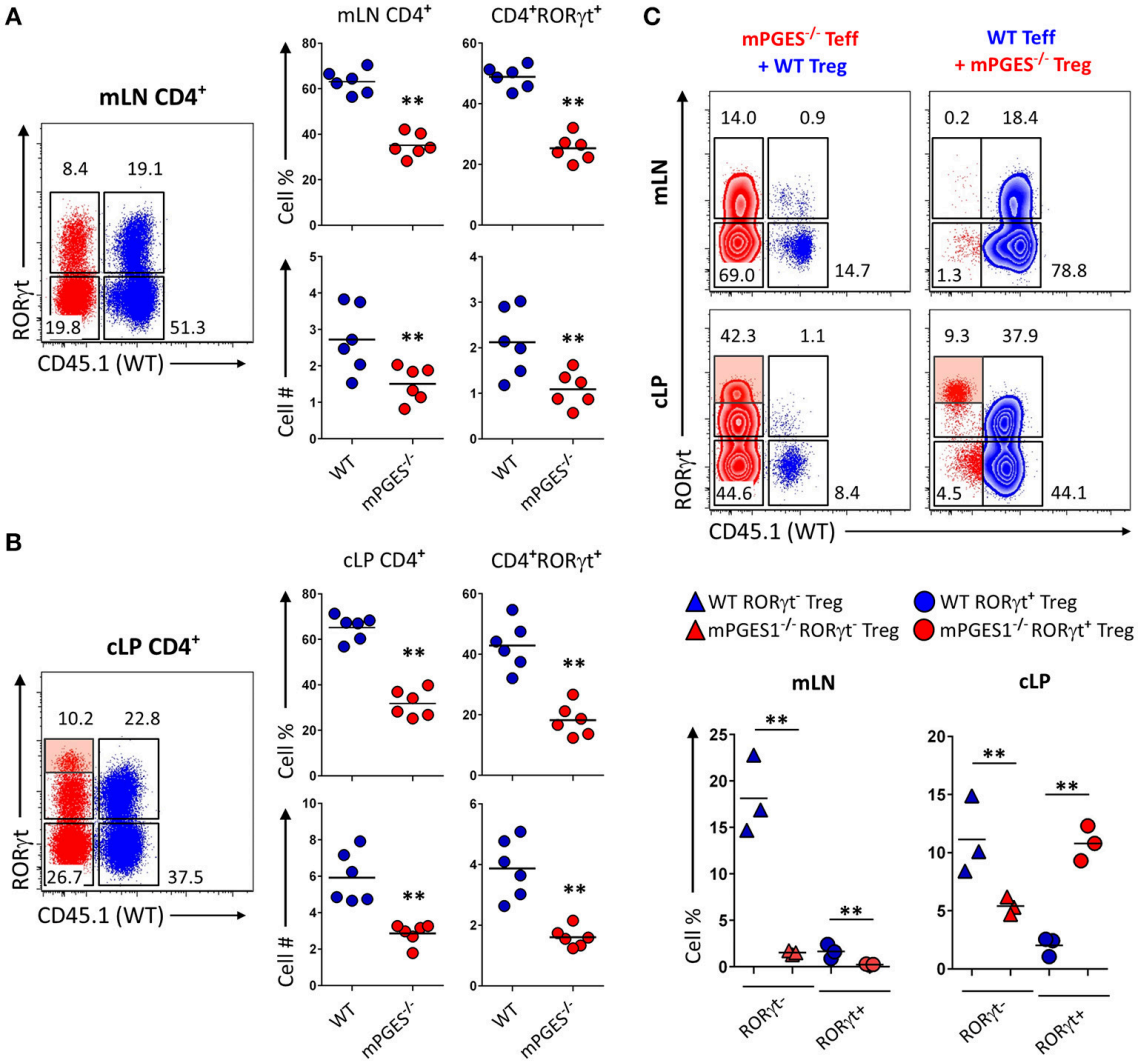
Deficiency in CD4-intrinsic mPGES-1 impairs Teff CD4^+^ cell expansion but enhances Treg localization and RORγt expression in the colonic lamina propria. **(A,B)** Rag1^−/−^ recipient mice received a co-transfer of a 1:1 mix of CD45.1^+^ WT (blue) and CD45.2^+^ mPGES-1^−/−^ (red) Teff cells. Flow cytometric analysis of the **(A)** mLN and **(B)** cLP CD4^+^ populations, with representative dot plots indicating intracellular expression of CD45.1 or CD45.2 congenic marker expression together with RORγt. In the cLP plot **(B)**, the shaded box indicates a unique RORγt^hi^ population of mPGES-1 deficient cells in the cLP. Graphs on the right indicate the proportions and total numbers for each group. **(C)** Co-transfer of either CD45.1^+^ WT Treg with CD45.2^+^ mPGES-1^−/−^ Teff cells or CD45.2^+^ mPGES-1^−−/−^ Treg cells with CD45.1^+^ WT Teff into Rag1^−/−^ recipients. Transfers were always performed with a 2:1 Teff:Treg ratio. In the cLP, mPGES-1^−/−^ CD4^+^ T cells are able to acquire higher RORγt expression than WT cells (shaded boxes). These CD4^+^RORγt^hi^ cells arise from both mPGES-1^−/−^ Teff cells and mature Treg cells. Graphs on the bottom show the proportions of WT or mPGES-1^−/−^ Treg cells that are either RORγt^−^ or RORγt^+^ in the mLN or the cLP. **P < 0.05 using in a one-way ANOVA with Welch's correction.

To determine if the reduced colitogenic potential observed in absence of mPGES-1 Teff was due to a reduced proliferation advantage, we co-transferred Teff cells from CD45.1^+^ WT and CD45.2^+^ mPGES-1^−/−^ animals in a 1:1 ratio into Rag1^−/−^ recipient animals. Ten weeks after transfer, the mLN (Figure 3A) and cLP (Figure 3B) were analyzed. Under these competitive circumstances, WT T cells were clearly able to expand more than T cells deficient in mPGES-1. This translated into significantly more CD4^+^RORγt^+^ cells derived from WT T cells. However, we observed that mPGES-1^−/−^ Teff CD4^+^ cells were capable of generating a proportion of RORγt^hi^ T cells in the LP that was absent in the WT donor T cells (Figure 3B, shaded box). It is known that colonic CD4^+^ cells display unique characteristics like high co-expression of RORγt in their FoxP3^+^ population (32, 33). This prompted us to investigate if mPGES-1 sufficiency would impact RORγt expression in Tregs in the colon. For this purpose, a mix of either CD45.1^+^ WT Teff and CD45.2^+^ mPGES-1^−/−^ Treg cells or a reciprocal CD45.1^+^ WT Treg and CD45.2^+^ mPGES-1^−/−^ Teff cells mix were injected in a 2:1 (Teff:Treg) ratio into Rag1^−/−^ recipient animals, and mLN and cLP were examined as previously described. Total WT Treg cells were found in much larger proportions than mPGES-1^−/−^ Treg in the mLN, independently of whether they expressed RORγt or not (Figure 3C). In stark contrast, mPGES-1^−/−^ Treg cells showed a much larger proportion of RORγt^+^ cells in the cLP. Furthermore, mPGES-1^−/−^ Treg cells showed greater relative expression levels of RORγt than WT Tregs in the cLP (Figure 3C shaded boxes). In summary, we demonstrate that mPGES-1 deficiency in T cells reduces their expansion capacity and colitogenic potential. Additionally, absence of T cell intrinsic mPGES-1 strongly promotes the localization of Tregs into the cLP to the detriment of the draining mLN, and this effect is simultaneous with acquisition of high expression levels of RORγt. These data suggest unique characteristics of mPGES-1 deficient T cells that protect from colitis.

### Inability to Sense PGE_2_ Through EP4 by CD4^+^ Effector T Cells Impairs Their Colitogenicity

**Figure 4:**
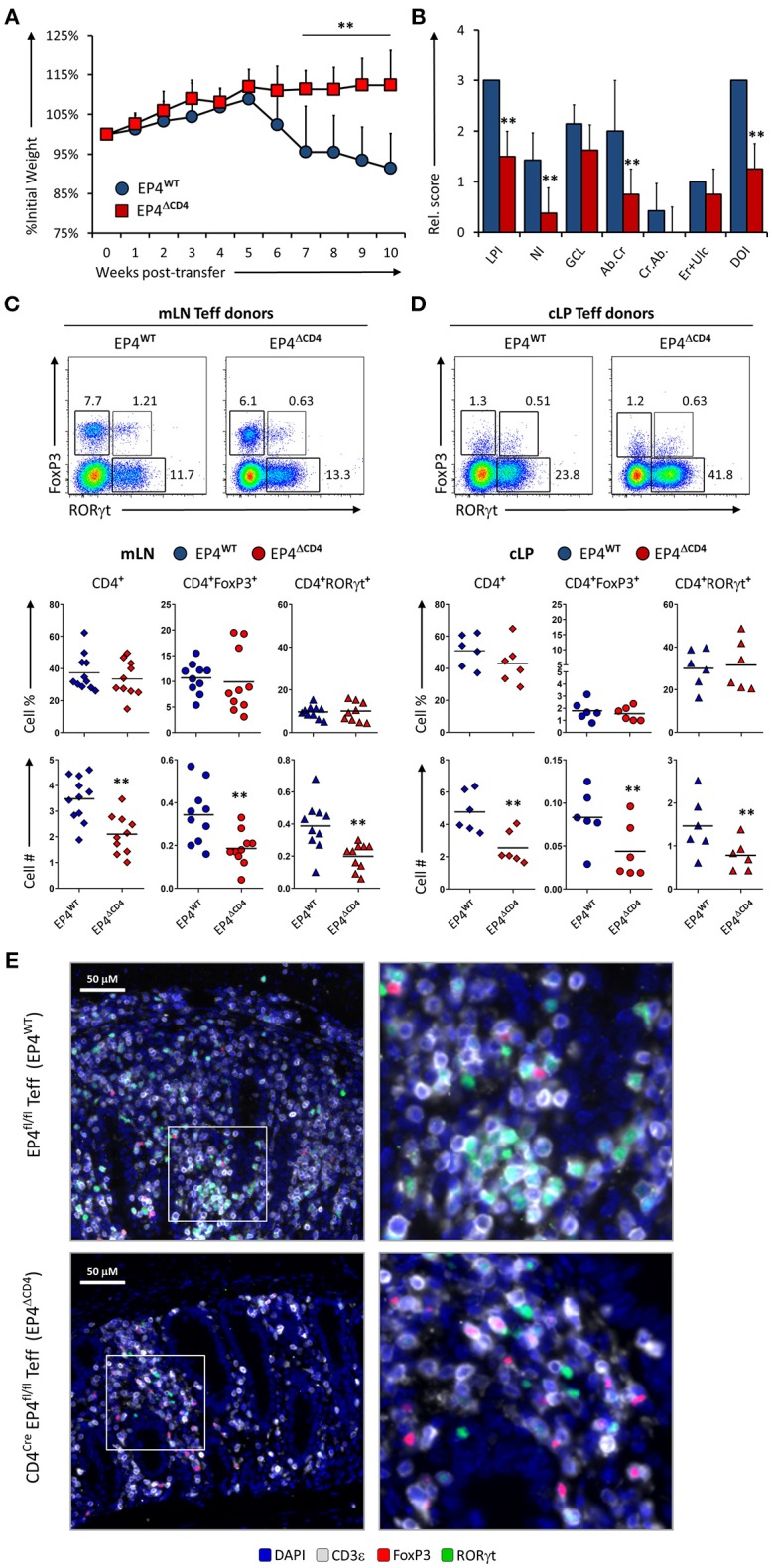
EP4 deficient CD4^+^ T effector cells have severely blunted colitogenic potential due to impaired proliferative capacity**. (A)** Weight loss in Rag1^−/−^ mice that received transfer of Teff cells from EP4^fl/fl^ (EP4^WT^) or CD4^Cre^ × EP4^fl/fl^ (EP4^Δ*CD*4^) donor mice. **(B)** Colon pathology scores from both cohorts. Flow cytometry analysis of the **(C)** mLN and **(D)** cLP CD4^+^ populations at the end of the experiment (week 10), indicating intracellular expression of RORγt and FoxP3 in summarized results from 4 experiments. **(E)** Colon images detailing lamina propria T cell infiltrates in both groups, with magnified inserts on the right-hand side. Blue = DAPI, Gray = CD3ε, Green = RORγt, and Red = FoxP3. **P < 0.01 using in a one-way ANOVA with Welch's correction.

Detection of PGE_2_ through EP4 increases Th1 responses when IL-12 is present (12) but also amplifies Th17 expansion in synergy with IL1β/IL-23 (10, 11). To examine if sensing of PGE_2_ produced during inflammation of the intestines was mediated through EP4 on T cells, we injected either EP4^fl/fl^ (virtual WT) and CD4^Cre^xEP4^fl/fl^ (EP4^Δ*CD*4^) Teff cells into Rag1^−/−^ recipient animals and evaluated colitis development and T cell phenotype as previously described. Mice that received Teff cells deficient in EP4 were significantly protected from colitis development, with little to no weight loss, and significantly less overall LP infiltration and neutrophils (Figures 4A,B). Examination of mLN and cLP infiltrates by microscopy and flow cytometry revealed a decrease in total CD4^+^ numbers that directly translated into less CD4^+^FoxP3^+^ and CD4^+^RORγt^+^ cells, but with no alteration of CD4^+^ subsets proportions (Figures 4C,D) on either examined anatomical location. Microscopic analysis of CD3ε, RORγt, and FoxP3 distribution within the cLP revealed that WT Teff cells accumulated in larger conglomerates compared to EP4^Δ*CD*4^ Teff cells (Figure 4E). We conclude that EP4 expression in CD4^+^ Teff cells is critical for their proliferative capacity without T cell subset specificity.

### Paracrine mPGES-1-deficiency in Non-lymphoid Cells Facilitates Colitis by Inhibiting Generation of CD4^+^FoxP3^+^ Cells

**Figure 5:**
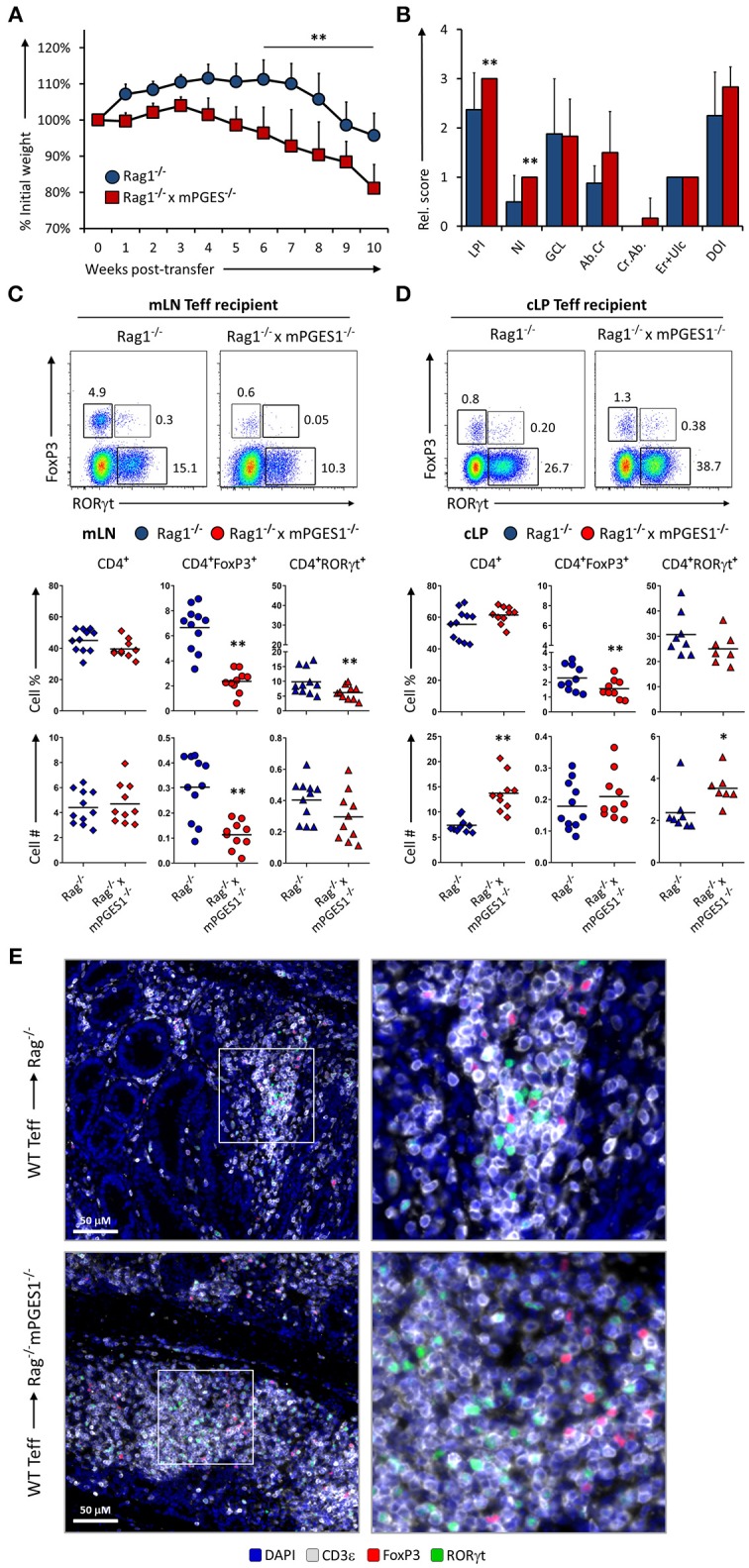
Paracrine mPGES-1-deficiency in non-lymphoid cells facilitates colitis by inhibiting generation of CD4^+^FoxP3^+^ cells**. (A)** Weight loss in Rag1^−/−^ or Rag1^−/−^ x mPGES-1^−/−^ mice that received a transfer of WT Teff donor cells. **(B)** Segregated colon pathology scores from both cohorts. Flow cytometry analysis of **(C)** mLN and **(D)** cLP CD4^+^ populations at the end of the experiment (10 weeks), indicating intracellular expression of RORγt and FoxP3 in summarized results from 3 experiments. **(E)** Fluorescence microscopy analysis of colon sections denoting CD4^+^ cell infiltrates. Blue = DAPI, Gray = CD3ε, Green = RORγt, and Red = FoxP3. **P < 0.01 and *P < 0.05 using in a one-way ANOVA with Welch's correction.

Non-lymphoid cells have the ability to produce large amounts of PGE_2_ in a COX-2-dependent manner (8, 13, 34). The net effect of PGE_2_ during intestinal inflammation is however hard to discern, as PGE_2_ has been reported to display both pro-inflammatory effects, and also result in protection from intestinal damage. We sought to determine whether production of PGE_2_ mediated by mPGES-1 in the cLP non-lymphoid cell compartment would alter T cell pathogenicity. For that purpose, we transferred WT Teff CD4^+^ cells into either Rag1^−/−^ or Rag1^−/−^ × mPGES-1^−/−^ recipients. Recipient mice lacking mPGES-1 developed colitis faster, lost more weight, and displayed more overall cell infiltration and neutrophilic content (Figures 5A,B). Analysis of the mLN and cLP of colitic mice revealed that absence of non-lymphoid mPGES-1 impaired the generation of CD4^+^FoxP3^+^ T cells in both tissues (Figures 5C–E), but more strikingly in the mLN where CD4^+^FoxP3^+^ T cell proportions and numbers were markedly decreased. The proportion of CD4^+^RORγt^+^ cells was also significantly reduced in absence of mPGES-1 in the mLN. However, in the cLP, total CD4^+^ and RORγt^+^CD4^+^ cells numbers were increased in the mPGES-1-deficient Rag1^−/−^ mice. Additionally, CD4^+^ cells from the cLP of Rag1^−/−^ × mPGES-1^−/−^ recipients were found to form more densely aggregated foci than in Rag1^−/−^ recipients (Figure 5E). We conclude that deficiency of mPGES-1 in non-lymphoid tissues enhances colitis by reducing the generation of CD4^+^FoxP3^+^ T cells in the mLN and increasing pathogenic CD4^+^ T cells in the cLP. These data suggest that paracrine mPGES-1-derived PGE_2_ may help to limit immune-mediated colitis by facilitating generation of Tregs in mLN and reducing pathogenic T cells in the cLP.

### PGE_2_ Controls Phosphorylation of STAT3 in Colon CD4^+^ Cells During Colitis

We have previously described that PGE_2_ can diminish phosphorylation of STAT3 during Th17 polarization (13). This led us to hypothesize that phosphorylation of STAT3 in colonic T cells could be altered by local PGE_2_. We quantified the numbers of pSTAT3^+^ cells in colon samples of our previous experiments to determine the relative contribution of either PGE_2_ signaling through EP4 (EP4^Δ*CD*4^, group 2) or host non-lymphoid mPGES-1-mediated PGE_2_ (Rag1^−/−^ × mPGES-1^−/−^ recipients, group 3). We focused on these groups as they displayed the biggest differences when compared to a standard colon pathogenic response induced by WT Teff (group 1).

**Figure 6:**
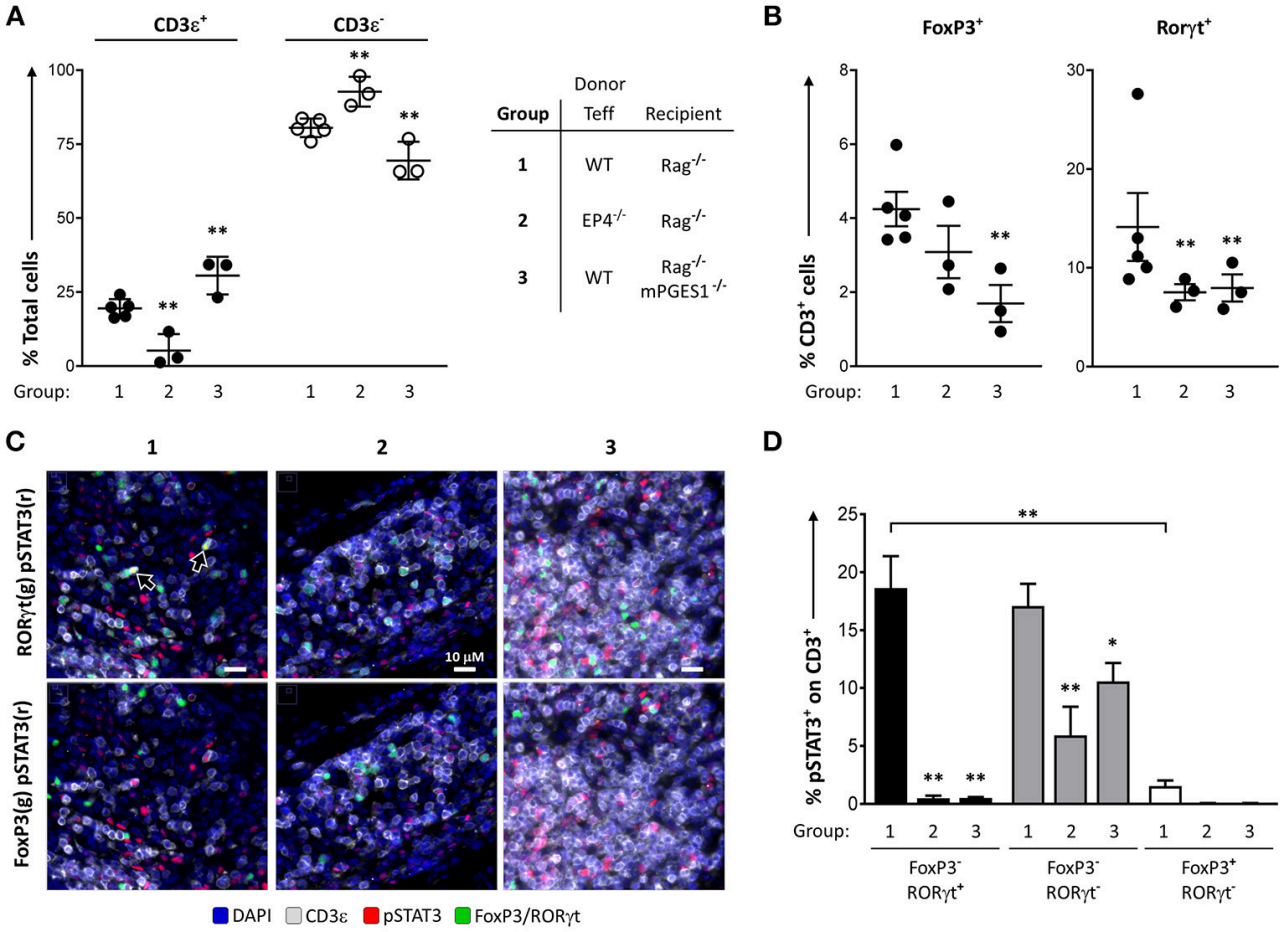
PGE_2_ controls phosphorylation of STAT3 in colon-infiltrating T cells. Individual colons from the 3 different groups of colitic mice indicated (1 = WT Teff → Rag1^−/−^, 2 = EP4^Δ*CD*4^ Teff → Rag1^−/−^, 3 = WT Teff → Rag1^−/−^ x mPGES-1^−/−^) were processed and stained for fluorescence microscopy evaluation DAPI, CD3ε, RORγt, FoxP3, and pSTAT3. **(A)** Quantification of total CD3ε^+^ and CD3ε^−^ cells in 15 regions/colon containing a total of 15–30K cells. **(B)** Quantification of total CD3ε^+^FoxP3^+^ and CD3ε^+^RORγt^+^ cells in the same groups. **(C)** Representative colon sections depicting co-localization of RORγt (green), pSTAT3 (red) and CD3 (gray) in the upper row, or of FoxP3 (green), pSTAT3 (red) with CD3 (gray) in the lower row in the same 3 groups. **(D)** Summary of the quantification of pSTAT3^+^ cells within the indicated CD3ε^+^ subsets (single RORγt^+^, RORγt^−^FoxP3^−^ or single FoxP3^+^). *P < 0.05 and **P < 0.01 using a one-way ANOVA with Welch's correction.

We first validated the quantification of different T cell populations by microscopy and computational analysis in whole colon tissue samples (Figures 6A,B). The percentages of CD3ε^+^ cells, as well as the CD3ε^+^FoxP3^+^, and CD3e^+^RORγt^+^ subsets, were consistent with the previous data observed by flow cytometry analysis of the cLP. Thus, the percentage of CD3ε^+^ cells was lowest when EP4 null cells were used as donor Teff and was highest when recipient mice were deficient in mPGES-1 (Figure 6A). Additionally, there were significantly lower CD3ε^+^FoxP3^+^ cell percentages in mPGES-1-deficient recipient mice compared to WT recipient mice, and lower CD3e^+^RORγt^+^ percentages with EP4 deficiency transferred Teff (Figure 6B). Of note, the percentages of CD3e^+^RORγt^+^ in the colon were not fully consistent when we compare the flow cytometry data (Figures 4C, 5C) with the results obtained with microscopy fluorescent quantification (Figure 6B). We explain this discrepancy due to a combination of differences in sample size (number of cells and coverage/colon), sample preparation-antigen reactivity, and technique (microscopy vs. flow cytometry). Co-expression of pSTAT3 was observed with RORγt (Figure 6C, arrowheads), but it was almost fully absent in FoxP3^+^ cells. Additionally, pSTAT3 expression was harder to detect in absence of EP4 on transferred Teff cells (group 2) or in absence of mPGES-1 in the recipient colon (group 3). We then quantified pSTAT3 positivity within each of the different T cell subsets that were previously evaluated in all the T cells present in 10 combined sections per colon (Figure 6D). WT (group 1) CD4^+^RORγt^+^ and RORγt- showed similar proportions of pSTAT3^+^ cells, while CD4^+^FoxP3^+^ cells showed a very reduced percentage comparatively (Figure 6D). Interestingly, both lack of T cell intrinsic EP4 deficiency and mPGES-1-derived PGE_2_ in the recipient colon niche abrogated almost all pSTAT3 in CD4^+^RORγt^+^ and CD4^+^FoxP3^+^ cells (black and white bars, Figure 6D). However, CD4^+^RORγt^−^FoxP3^−^ cells exhibited different sensitivities to PGE_2_: Inability to detect PGE_2_ with EP4 decreased phosphorylation of STAT3 by more than 50% (group 2), while absence of paracrine PGE_2_ more modestly reduced pSTAT3 percentages (group 3) (gray bars, Figure 6D). We conclude that upregulation of pSTAT3 in colonic T cells is strongly influenced by PGE_2_, and abrogating either EP4 sensing or paracrine PGE_2_ production inhibits STAT3 phosphorylation.

## Discussion

This study is the first to demonstrate the divergent effects of T cell-derived PGE_2_ compared with non-lymphocyte-derived PGE_2_ on T cell phenotypes during colitis. Using a T cell dependent model of colitis, we show that deficient production or sensing of PGE_2_ reduces the colitogenic potential of transferred CD4^+^ T cells. Deficient production of PGE_2_ due to deletion of mPGES-1 in transferred T cells reduces colitis in association with developing CD4^+^ cells expressing high levels of RORγt. Deficient sensing by deletion of the EP4 receptor almost completely abolishes clinical colitis likely due to reduced T cell proliferation in the colonic lamina propria. In contrast, transfer of WT T effector cells into recipient mice deficient in mPGES-1 results in a marked increase in colitogenesis. mPGES-1 deficiency in non-lymphoid cells impairs the development of FoxP3^+^ cells in mLN and increases infiltration of total CD4^+^ cells in the cLP. This finding suggests that PGE_2_ is required for proper development of Th17 and Treg cells that restrain colitis.

PGE_2_ has been classically regarded as a driver and perpetuator of inflammation (2, 16, 35), but it is also clear that it has potent protective roles (36). In the intestines, the COX1, COX2, and mPGES-1 enzymes control PGE_2_ levels, but they differ in their tissue expression pattern. Indeed, indiscriminate inhibition of COX enzymatic activity with piroxicam accelerates colitis in both IL10^−/−^ mice (37) and during transfer of CD4^+^ cells from IL10^−/−^ mice into Rag1^/−^ mice (38). However, the role of mPGES-1 during intestinal inflammation has not been as deeply studied. mPGES-1-deficient animals are partially protected from dextran sulfate sodium (DSS)-induced colitis (35), and PGE_2_ treatment alleviates mucosal injury and induces EP4 expression during DSS-induced colitis in WT mice (39). These data suggest a protective role of mPGES-1-derived PGE_2_ during intestinal injuries, but the mechanisms describing how mPGES-1 competence on tissue damaging cells like lymphocytes affects colitis is unknown. In the current study we show that in absence of inflammatory stimuli, nearly 50% of production of PGE_2_ in colon explants relies on mPGES-1 competence (Figure 1A). Lymphocytes present in the colon contribute less dramatically to this basal PGE_2_ pool, but colons void of lymphocytes that also lack mPGES-1 (Rag1^−/−^ × mPGES-1^−/−^) show further reduced PGE_2_ concentrations, indicating some synergy of environmental mPGES-1 expression with lymphocytes. A significant proportion of this basal PGE_2_ can be inhibited by specifically inhibiting COX-2 when either mPGES-1 and/or lymphocytes are present, but not when colons lack both components.

Dysregulation of T cell activity during colitis leads to harmful responses incapacitating Tregs at the same time that pathogenic Th1 and Th17 responses are heightened (40, 41). The pathogenic potential of Th1 and Th17 cells actually depends on the colitis model studied (42, 43), and it has recently been shown that IFNγ production is necessary in Th17 cells to cause colitis (24). Indeed, although Th17 cells are generally associated with a pro-inflammatory response in autoimmunity, IL-17A can have very different effects during colitis (44, 45). The potential protective function of Th17-driven IL-17A in IBD is also exemplified by the recent failure of mAb therapies that target IL-17 such as secukinumab (46). In this study, we show that T cell intrinsic mPGES-1 deficiency confers partial protection from colitis development, and this is associated with increased RORγt-expressing cells in the mLN and cLP (Figures 2C–E). Additionally, mPGES-1 deficiency also impairs the capacity of CD4^+^ Teff cells to more efficiently expand compared to WT CD4^+^ Teff cells (Figures 3A,B). This aspect of T cell biology contrasts with the lack of T cell-intrinsic mPGES-1 effect that we recently found during a proinflammatory T cell responses in a mouse model of collagen-induced arthritis (CIA) (13). These differences are likely due to several factors related to the characteristics of each model. Most of the T cell expansion in a Rag-deficient mouse are due to homeostatic expansion that is not controlled by Tregs, while the CIA model involves a more moderate and antigen-specific and adjuvant-boosted expansion. Furthermore, the T cell responses we observed in the CIA model happened early on, while T cell homeostatic expansion in the Rag-deficient model takes weeks to develop, and additionally, the specific anatomical locations where expansion occurs are very different (draining lymph nodes vs. lamina propria). Interestingly, we found that the frequencies of CD4^+^FoxP3^+^ cells were consistently reduced in the cLP compared to the mLN when only Teff cells were transferred, while CD4^+^RORγt^+^ cells were generally increased, as it would be expected by a preferential microbiota-driven migration and expansion of Th17 cells in the intestines. It is hence important to reflect on the fact that T cell expansion can strongly vary due to the distinct inflammatory conditions and microenvironment-imposed constraints, and thus the impact of local concentrations of PGE_2_ mediated by mPGES-1 or other enzymes contributing to the PGE_2_ pool can also render different results.

Our results demonstrate the paradoxical finding that mPGES-1^−/−^ Teff cells are partially protective compared to WT Teff, associated with an increase in CD4^+^RORγt^+^ cells (Figure 2), while EP4^−/−^ Teff cells also exert a protective effect, but in this case with a decrease in CD4^+^RORγt^+^ cells (Figure 4). Whether the phenotype or the expansion of T cells are more affected by PGE_2_ and how this is related to colitis is a complex question. PGE_2_ sensing through EP4 controls *in vivo* expansion of the total numbers of T cells. This is a straightforward result that we interpret as related to T cell numbers regardless of their phenotype. mPGES-1-mediated PGE_2_ production has differing effects depending on whether the source is autocrine, examined here by transfer of mPGES-1-deficient Teff cells (Figures 2, 3), or paracrine, examined here using Rag1 and mPGES-1 double deficient recipients of WT Teff cells (Figures 5, 6). Autocrine T cell intrinsic PGE_2_ production appears to control the balance of T cell phenotypes while paracrine PGE_2_ appears to be protective of colitis and may facilitate development of Treg cells. Therefore, depending on the specific inflammatory response and location, PGE_2_ can induce T cell expansion through EP4 but also control intestinal damage by altering Th17 and Treg phenotypes. The effect of PGE_2_ on T cell phenotype is likely to be complex and dependent on the overall inflammatory milieu. Our results also point to the plastic nature of Th17 and Treg responses, and how these classically defined phenotypes should be constantly re-evaluated based on context.

mPGES1^−/−^ CD4^+^ Teff and Treg cells can acquire higher levels of RORγt expression exclusively in the cLP (Figures 3B,C), a phenotype that was especially apparent when mPGES-1-deficient Tregs were transferred (Figure 3C). It has previously been shown that a proportion of intestinal FoxP3^+^ Treg cells co-express RORγt, and their presence is dependent on intestinal microbiota (33, 47). The suppressive capacities of CD4^+^FoxP3^+^RORγt^+^ cells have been reported to be superior to their FoxP3^+^RORγt^−^ counterparts (32), and generally geared toward controlling intestinal Th2 and Th17 responses (48, 49). It is tempting to speculate that the RORγt^hi^ cells we observed might be responsible for the reduction in clinical colitis when mPGES-1-deficient cells were transferred. STAT3 is necessary for the Th17 lineage to develop (48), and is also a master regulator of Treg and Th17 lineage commitment (50). STAT3 phosphorylation mediates resistance of human T cells to regulatory T cell suppression (51). We have previously reported that exogenous addition of PGE_2_ can downregulate pSTAT3 of CD4^+^ cells during Th17 polarization *in vitro* (13). We now show that during the late phases of induced colitis, phosphorylation of STAT3 is present on nearly 20% of all cLP CD3^+^FoxP3^−^RORγt^+^ and CD3^+^FoxP3^−^RORγt^−^ cells (Figures 6C,D), while it is barely expressed in CD3^+^FoxP3^+^ RORγt^−^ regulatory T cells. Moreover, ablation of mPGES-1 in recipient mice or EP4 expression in Teff cells dramatically reduced pSTAT3 signal specifically in CD3^+^FoxP3^−^RORγt^+^ cells, while this reduction was significant but more modest in the CD3^+^FoxP3^−^RORγt^−^ compartment. These data imply that the context of exposure to different PGE_2_ levels and signals is critically important for phosphorylation of STAT3 in different T cell populations.

We have also identified that deficiency of paracrine mPGES1-driven PGE_2_ exacerbates T cell-driven colitis by selectively decreasing CD4^+^FoxP3^+^ cells in the mLN (Figure 5C), with a concomitant increase of total number of CD4^+^ infiltration in the cLP (Figure 5D). Classical *ex vivo* suppression assays with WT or mPGES-deficient Treg showed no difference in suppression capacity (Figure S1) suggesting that effects of PGE_2_ are related to changes in differentiation to Treg or migration to the colon. Additionally, no differences in disease progression were found when comparing WT and mPGES1-deficient Treg-intrinsic suppressive capacity *in vivo* (Figure 2A, discontinuous lines). The specific effects that we observed in mLN or cLP due to mPGES1-derived PGE_2_ in recipient mice could be explained by differences in gut tropism, which we are currently investigating. In this regard, PGE_2_ has been shown to inhibit the production of retinoic acid by intestinal CD103^+^ DCs and their capacity to express CCR9 upon T cell priming (52).

Signaling through EP2 can induce proliferation and cytokine secretion in Th17 cells (10), but human Th17 cells show specific downregulation of EP2 expression through RORC-dependent silencing (6). In the context of IBD, certain polymorphisms have been found in patients with Crohn's Disease that lead to increased expression of EP4 (53). From all EPs, only EP4 is critical to prevent mucosal damage in murine colitis induced with DSS (5). EP4 signaling is also critical for *in vitro* generation of Th1 cells and for expansion of Th17 cells upon IL-23 exposure (11), but surprisingly EP4 is downregulated during murine Th17 polarization in absence of IL-23 (13). Hemizigosity in EP4 during T cell induced colitis in the adoptive T cell transfer colitis model is partially protective, and also affects IFNγ and IL2 production by MLN CD4^+^ cells (12). In our study we show that EP4 controls expansion of all phenotypes derived from Teff transfers during colitis (Figure 4), and therefore EP4-deficiency confers protection.

Th17 cell generation relies on sensing IL-23 signals generated during intestinal inflammation (54), and IL-23 also restrains Treg cells (55). PGE_2_ can stimulate IL-23 production by DCs, myeloid cells, and other cells present in the intestines (2, 10, 36). It is hence possible that PGE_2_ potentiates IL-23-mediated intestinal inflammation through enhancing EP4-dependent pathogenic CD4^+^RORγt^+^ responses, while in other instances and locations (like the mLN) it can induce CD4^+^FoxP3^+^ cells that protect from colitis development. However, Treg and Th17 cell lineage commitment can display significant plasticity, a feature especially patent in mucosal sites (24, 56, 57). In this context, PGE_2_ could act inducing shifts in T cell lineage commitment, either directly on T cells or by altering the cytokine milieu generated by surrounding APCs: PGE_2_ could hence act in mature Tregs by contributing to acquire FoxP3 expression in first place, but also to gain RORγt expression on FoxP3^+^ cells. PGE_2_ can also inhibit IL-17A and induce IFNγ production *in vitro* during Th17 polarization (13), so a different effect of PGE_2_ could be its impact on promoting IFNγ in effector T cells.

In summary, we provide evidence that supports both pro- and anti-inflammatory effects of PGE_2_ on T cells and colitis. We show that PGE_2_ can exert opposite effects on T cell colitogenicity depending on the source of such PGE_2_ and how it is sensed. Our results imply that extreme caution should be considered when using drugs that modulate PGE_2_ production in a non-cell specific manner, as they can have disparate net effects. Our research also suggests that cell specificity and spatio-temporal considerations of PGE_2_ production within the colon can be exploited to promote regulatory vs. pathogenic T cell function.

## Author Contributions

DM, KL, and LC were responsible for experimental design, data interpretation, and manuscript preparation. DM, AB, and EJ performed the experiments. DM, AB, HK, KL, and LC contributed to data analysis, MW performed the patho-histological scoring. DM and AB performed the preparation, labeling, and imaging of colon tissues. AB and HK performed the colon fluorescent signal quantifications.

### Conflict of Interest Statement

The authors declare that the research was conducted in the absence of any commercial or financial relationships that could be construed as a potential conflict of interest.

## Supplementary Material

Figure S1 *In vitro* T reg suppression assays with WT and mPGES-1-deficient Tregs Conventional CD4^+^ cells (Tconv, CD4^+^CD25^−^) were cocultured with either WT or mPGES1-deficient Tregs (Treg, CD4^+^CD25^+^) isolated and pooled from 3 different mice in the presence of plate bound anti-CD3ε (0.5 mg/ml) and soluble anti-CD28 (0.5 mg/ml) for 4 days in different Tconv:Treg ratios to address the Treg suppressive capacities. **(A)** Representative histogram depicting a CFSE dilution assay to evaluate Tconv proliferation in presence of either WT or mPGES-1-deficient Treg cells (1:2 Tconv:Treg ratio) and Tconv alone. **(B)** Summary of proliferating percentages at different Tconv:Treg ratios. Shown is one of two representative experiments.

## References

[1] Park JY Pillinger MH Abramson SB. Prostaglandin E2 synthesis and secretion: the role of PGE2 synthases. Clin Immunol. (2006) 119:229–40. 10.1016/j.clim.2006.01.016 16540375

[2] Kalinski P. Regulation of immune responses by prostaglandin E2. J Immunol. (2012) 188:21–8. 10.4049/jimmunol.1101029 22187483 PMC3249979

[3] Williams CS Mann M DuBois RN. The role of cyclooxygenases in inflammation, cancer, and development. Oncogene (2000) 18:7908–16. 10.1038/sj.onc.1203286 10630643

[4] Dey I Lejeune M Chadee K. Prostaglandin E2 receptor distribution and function in the gastrointestinal tract. Br J Pharmacol. (2006) 149:611–23. 10.1038/sj.bjp.0706923 17016496 PMC2014644

[5] Kabashima K Saji T Murata T Nagamachi M Matsuoka T Segi E. The prostaglandin receptor EP4 suppresses colitis, mucosal damage and CD4 cell activation in the gut. J Clin Invest. (2002) 109:883–93. 10.1172/JCI0214459 11927615 PMC150928

[6] Kofler DM Marson A Dominguez-Villar M Xiao S Kuchroo VK Hafler DA. Decreased RORC-dependent silencing of Prostaglandin receptor EP2 induces autoimmune Th17 cells. J Clin Invest. (2014) 124:2513–22. 10.1172/JCI72973 24812667 PMC4089462

[7] Muthuswamy R Mueller-Berghaus J Haberkorn U Reinhart TA Schadendorf D Kalinski P PGE2 transiently enhances DC expression of CCR7 but inhibits the ability of DCs to produce CCL19 and attract naive T cells. Blood (2010) 116:1454–9. 10.1182/blood-2009-12-258038 20498301 PMC2938836

[8] Monrad SU Kojima F Kapoor M Kuan EL Sarkar S Randolph GJ Genetic deletion of mPGES-1 abolishes PGE2 production in murine dendritic cells and alters the cytokine profile, but does not affect maturation or migration. Prostaglandins Leukot Essent Fat Acids (2011) 84:113–21. 10.1016/j.plefa.2010.10.003 PMC3072052 21190819

[9] Duffin R OConnor RA Crittenden S Forster T Yu C Zheng X Prostaglandin E2 constrains systemic inflammation through an innate lymphoid cell-IL-22 axis. Science (2016) 351:1333–8. 10.1126/science.aad9903 26989254 PMC4841390

[10] Boniface K Bak-Jensen KS Li Y Blumenschein WM McGeachy MJ McClanahan TK. Prostaglandin E2 regulates Th17 cell differentiation and function through cyclic AMP and EP2/EP4 receptor signaling. J Exp Med. (2009) 206:535–48. 10.1084/jem.20082293 19273625 PMC2699124

[11] Yao C Sakata D Esaki Y Li Y Matsuoka T Kuroiwa K. Prostaglandin E2–EP4 signaling promotes immune inflammation through TH1 cell differentiation and TH17 cell expansion. Nat Med. (2009) 15:633–40. 10.1038/nm.1968 19465928

[12] Yao C Hirata T Soontrapa K Ma X Takemori H Narumiya S Prostaglandin E2 promotes Th1 differentiation via synergistic amplification of IL-12 signalling by cAMP and PI3-kinase. Nat Commun. (2013) 4:1685 10.1038/ncomms2684 23575689 PMC3644078

[13] Maseda D Johnson EM Nyhoff LE Baron B Kojima F Wilhelm AJ. mPGES1-dependent Prostaglandin E2 (PGE2) controls antigen-specific Th17 and Th1 responses by regulating T autocrine and paracrine PGE2 production. J Immunol. (2017) 200:725–36. 10.4049/jimmunol.1601808 29237778 PMC5760456

[14] Napolitani G Acosta-Rodriguez EV Lanzavecchia A Sallusto F. Prostaglandin E2 enhances Th17 responses via modulation of IL-17 and IFN-ɤ production by memory CD4^+^ T cells. Eur J Immunol. (2009) 39:1301–12. 10.1002/eji.200838969 19384872

[15] Fabricius D Neubauer M Mandel B Schütz C Viardot A Vollmer A. Prostaglandin E2 inhibits IFN-alpha secretion and Th1 costimulation by human plasmacytoid dendritic cells via E-prostanoid 2 and E-prostanoid 4 receptor engagement. J Immunol. (2010) 184:677–84. 10.4049/jimmunol.0902028 20018632

[16] Nakanishi M Rosenberg DW. Multifaceted roles of PGE2 in inflammation and cancer. Semin Immunopathol. (2013) 35:123–37. 10.1007/s00281-012-0342-8 22996682 PMC3568185

[17] Schumacher Y Aparicio T Ourabah S Baraille F Martin A Wind P. Dysregulated CRTC1 activity is a novel component of PGE2 signaling that contributes to colon cancer growth. Oncogene (2016) 35:2602–14. 10.1038/onc.2015.283 26300003

[18] Sasaki Y Nakatani Y Hara S. Role of microsomal prostaglandin E synthase-1 (mPGES-1)-derived prostaglandin E2 in colon carcinogenesis. Prostaglandins Other Lipid Mediat. (2015) 121:42–5. 10.1016/j.prostaglandins.2015.06.006 26150361

[19] Vong L Ferraz JGP Panaccione R Beck PL Wallace JL A pro-resolution mediator, prostaglandin D2, is specifically up-regulated in individuals in long-term remission from ulcerative colitis. Proc Natl Acad Sci USA. (2010) 107:12023–7. 10.1073/pnas.1004982107 20547854 PMC2900663

[20] Long MD Kappelman MD Martin CF Chen W Anton K Sandler RS. Role of nonsteroidal anti-inflammatory drugs in exacerbations of inflammatory bowel disease. J Clin Gastroenterol. (2016) 50:152–6. 10.1097/MCG.0000000000000421 26485106 PMC4703528

[21] Ishikawa TO Oshima M Herschman HR Cox-2 deletion in myeloid and endothelial cells, but not in epithelial cells, exacerbates murine colitis. Carcinogenesis (2011) 32:417–26. 10.1093/carcin/bgq268 21156970 PMC3047239

[22] Roulis M Nikolaou C Kotsaki E Kaffe E Karagianni N Koliaraki V. Intestinal myofibroblast-specific Tpl2-Cox-2-PGE2 pathway links innate sensing to epithelial homeostasis. Proc Natl Acad Sci USA. (2014) 111:E4658–67. 10.1073/pnas.1415762111 25316791 PMC4217397

[23] Wang Z Friedrich C Hagemann SC Korte WH Goharani N Cording S. Regulatory T cells promote a protective Th17-associated immune response to intestinal bacterial infection with C. rodentium. Mucosal Immunol. (2014) 7:1290–301. 10.1038/mi.2014.17 24646939

[24] Harbour SN Maynard CL Zindl CL Schoeb TR Weaver CT. Th17 cells give rise to Th1 cells that are required for the pathogenesis of colitis. Proc Natl Acad Sci USA. (2015) 112:7061–6. 10.1073/pnas.1415675112 26038559 PMC4460486

[25] Kempski J Brockmann L Gagliani N Huber S. TH17 cell and epithelial cell crosstalk during inflammatory bowel disease and carcinogenesis. Front Immunol. (2017) 8:1373. 10.3389/fimmu.2017.01373 29118756 PMC5660962

[26] Trebino CE Stock JL Gibbons CP Naiman BM Wachtmann TS Umland JP. Impaired inflammatory and pain responses in mice lacking an inducible prostaglandin E synthase. Proc Natl Acad Sci USA. (2003) 100:9044–9. 10.1073/pnas.1332766100 12835414 PMC166435

[27] Schneider A Guan YF Zhang Y Magnuson MA Pettepher C Loftin CD. Generation of a conditional allele of the mouse prostaglandin EP4receptor. Genesis (2004) 40:7–14. 10.1002/gene.20048 15354288

[28] Weigmann B Tubbe I Seidel D Nicolaev A Becker C Neurath MF. Isolation and subsequent analysis of murine lamina propria mononuclear cells from colonic tissue. Nat Protoc. (2007) 2:2307–11. 10.1038/nprot.2007.315 17947970

[29] Gerdes MJ Sevinsky CJ Sood A Adak S Bello MO Bordwell A. Highly multiplexed single-cell analysis of formalin-fixed, paraffin-embedded cancer tissue. Proc Natl Acad Sci USA. (2013) 110:11982–7. 10.1073/pnas.1300136110 23818604 PMC3718135

[30] McKinley ET Sui Y Al-Kofahi Y Millis BA Tyska MJ Roland JT. Optimized multiplex immunofluorescence single-cell analysis reveals tuft cell heterogeneity. JCI Insight (2017) 2:93487. 10.1172/jci.insight.93487 28570279 PMC5453701

[31] Kirkby NS Chan MV Zaiss AK Garcia-Vaz E Jiao J Berglund LM. Systematic study of constitutive cyclooxygenase-2 expression: role of NF-κB and NFAT transcriptional pathways. Proc Natl Acad Sci USA. (2016) 113:434–9. 10.1073/pnas.1517642113 26712011 PMC4720301

[32] Yang B-H Hagemann S Mamareli P Lauer U Hoffmann U Beckstette M Foxp3+ T cells expressing RORγt represent a stable regulatory T-cell effector lineage with enhanced suppressive capacity during intestinal inflammation. Mucosal Immunol. (2015) 205:1381–93. 10.1038/mi.2015.74 26307665

[33] Solomon BD Hsieh C-S. Antigen-specific development of mucosal Foxp3+RORγt+ T cells from regulatory T cell precursors. J Immunol. (2016) 197:3512–9. 10.4049/jimmunol.1601217 27671109 PMC5101183

[34] Newson J Motwani MP Kendall AC Nicolaou A Muccioli GG Alhouayek M. Inflammatory resolution triggers a prolonged phase of immune suppression through COX-1/mPGES-1-derived Prostaglandin E2. Cell Rep. (2017) 20:3162–75. 10.1016/j.celrep.2017.08.098 28954232 PMC5639146

[35] Montrose DC Nakanishi M Murphy RC Zarini S McAleer JP Vella AT The role of PGE2 in intestinal inflammation and tumorigenesis. Prostaglandins Other Lipid Mediat. (2015) 116–7:26–36. 10.1016/j.prostaglandins.2014.10.002 PMC4385488 25460828

[36] Chinen T Komai K Muto G Morita R Inoue N Yoshida H. Prostaglandin E2 and SOCS1 have a role in intestinal immune tolerance. Nat Commun. (2011) 2:190. 10.1038/ncomms1181 21304519 PMC3105338

[37] Holgersen K Kvist PH Markholst H Hansen AK Holm TL. Characterisation of enterocolitis in the piroxicam-accelerated interleukin-10 knock out mouse - A model mimicking inflammatory bowel disease. J Crohns Colitis (2014) 8:147–60. 10.1016/j.crohns.2013.08.002 23994255

[38] Blum AM Metwali A Elliott DE Berg DJ Weinstock JV. CD4+ T cells from IL-10-deficient mice transfer susceptibility to NSAID-induced Rag colitis. Am J Physiol Gastrointest Liver Physiol. (2004) 287:G320–5. 10.1152/ajpgi.00527.2003 15246967

[39] Peng X Li J Tan S Xu M Tao J Jiang J. COX-1/PGE2/EP4 alleviates mucosal injury by upregulating β-arr1-mediated Akt signaling in colitis. Sci Rep. (2017) 7:1055. 10.1038/s41598-017-01169-6 28432343 PMC5430694

[40] Shale M Schiering C Powrie F. CD4+ T-cell subsets in intestinal inflammation. Immunol Rev. (2013) 252:164–82. 10.1111/imr.12039 23405904 PMC3736165

[41] Kanai T Mikami Y Sujino T Hisamatsu T Hibi T. RORγt-dependent IL-17A-producing cells in the pathogenesis of intestinal inflammation. Mucosal Immunol. (2012) 5:240–7. 10.1038/mi.2012.6 22354322

[42] Giles EM Sanders TJ McCarthy NE Lung J Pathak M MacDonald TT. Regulation of human intestinal T-cell responses by type 1 interferon-STAT1 signaling is disrupted in inflammatory bowel disease. Mucosal Immunol. (2016) 10:184–93. 10.1038/mi.2016.44 27220814

[43] Zimmermann J Kühl AA Weber M Grün JR Löffler J Haftmann C. T-bet expression by Th cells promotes type 1 inflammation but is dispensable for colitis. Mucosal Immunol. (2016) 9:1487–99. 10.1038/mi.2016.5 26883725

[44] Lee JS Tato CM Joyce-Shaikh B Gulan F Cayatte C Chen Y. Interleukin-23-independent IL-17 production regulates intestinal epithelial permeability. Immunity (2015) 43:727–38. 10.1016/j.immuni.2015.09.003 26431948 PMC6044435

[45] Maxwell JR Zhang Y Brown WA Smith CL Byrne FR Fiorino M. Differential roles for interleukin-23 and interleukin-17 in intestinal immunoregulation. Immunity (2015) 43:739–50. 10.1016/j.immuni.2015.08.019 26431947

[46] Neurath MF Current and emerging therapeutic targets for IBD. Nat Rev Gastroenterol Hepatol. (2017) 14:269–78. 10.1038/nrgastro.2016.208 28144028

[47] Lochner M Peduto L Cherrier M Sawa S Langa F Varona R. *In vivo* equilibrium of proinflammatory IL-17+ and regulatory IL-10+ Foxp3+ RORγ t+ T cells. J Exp Med. (2008) 205:1381–93. 10.1084/jem.20080034 18504307 PMC2413035

[48] Ohnmacht C Park J Cording S Wing JB Atarashi K Obata Y. The microbiota regulates type 2 immunity through RORγt+ T cells. Science (2015) 349:989–93. 10.1126/science.aac4263 26160380

[49] Sefik E Geva-Zatorsky N Oh S Konnikova L Zemmour D McGuire AM. MUCOSAL IMMUNOLOGY. Individual intestinal symbionts induce a distinct population of RORγ(+) regulatory T cells. Science (2015) 349:993–7. 10.1126/science.aaa9420 26272906 PMC4700932

[50] Chaudhry A Rudra D Treuting P Samstein RM Liang Y Kas A. CD4+ regulatory T cells control TH17 responses in a Stat3-dependent manner. Science (2009) 326:986–91. 10.1126/science.1172702 19797626 PMC4408196

[51] Goodman WA Young AB McCormick TS Cooper KD Levine AD. Stat3 phosphorylation mediates resistance of primary human T cells to regulatory T cell suppression. J Immunol. (2011) 186:3336–45. 10.4049/jimmunol.1001455 21307288 PMC3133678

[52] Stock A Booth S Cerundolo V. Prostaglandin E2 suppresses the differentiation of retinoic acid–producing dendritic cells in mice and humans. J Exp Med. (2011) 208:761–73. 10.1084/jem.20101967 21444662 PMC3135350

[53] Prager M Büttner J Büning C. PTGER4 modulating variants in Crohn's disease. Int J Colorectal Dis. (2014) 29:909–15. 10.1007/s00384-014-1881-3 24793213

[54] Krausgruber T Schiering C Adelmann K Harrison OJ Chomka A Pearson C. T-bet is a key modulator of IL-23-driven pathogenic CD4+ T cell responses in the intestine. Nat Commun. (2016) 7:11627. 10.1038/ncomms11627 27193261 PMC4874038

[55] Ahern PP Izcue A Maloy KJ Powrie F. The interleukin-23 axis in intestinal inflammation. Immunol Rev. (2008) 226:147–59. 10.1111/j.1600-065X.2008.00705.x 19161422

[56] Miyao T Floess S Setoguchi R Luche H Fehling HJ Waldmann H Plasticity of Foxp3+T cells reflects promiscuous Foxp3 expression in conventional T cells but not reprogramming of regulatory T cells. Immunity (2012) 36:262–75. 10.1016/j.immuni.2011.12.012 22326580

[57] Gagliani N Vesely MCA Iseppon A Brockmann L Xu H Palm NW. Th17 cells transdifferentiate into regulatory T cells during resolution of inflammation. Nature (2015) 523:221–5. 10.1038/nature14452 25924064 PMC4498984

