# Supplementary figures and images for: mPGES-1-Mediated Production of PGE2 and EP4 Receptor Sensing Regulate T Cell Colonic Inflammation
Source: Front Immunol. 2018 Dec 14;9:2954. doi: 10.3389/fimmu.2018.02954 (PMC6302013; doi:10.3389/fimmu.2018.02954)

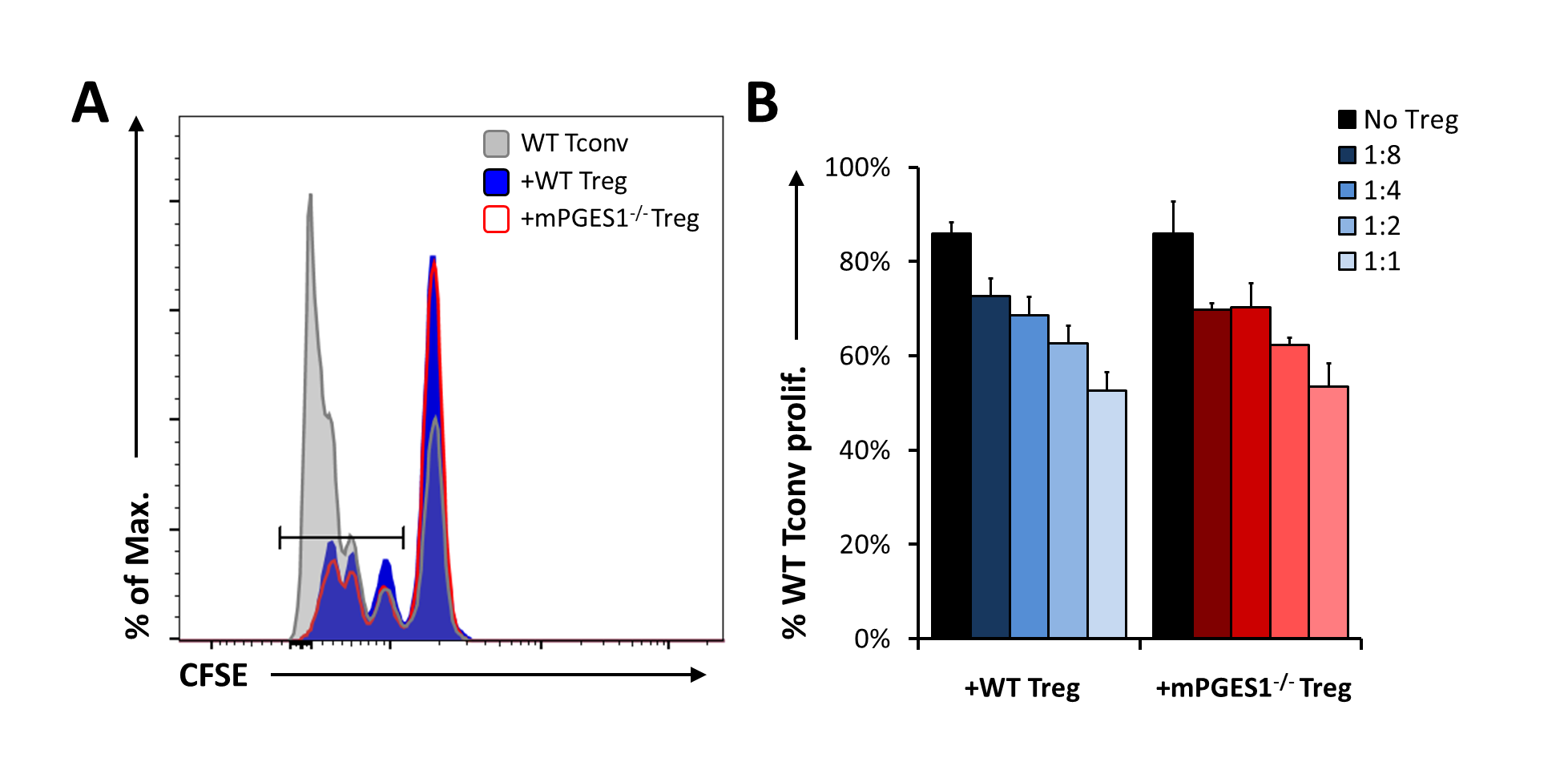

Supplement: Figure S1 — In vitro T reg suppression assays with WT and mPGES-1-deficient Tregs Conventional CD4+ cells (Tconv, CD4+CD25−) were cocultured with either WT or mPGES1-deficient Tregs (Treg, CD4+CD25+) isolated and pooled from 3 different mice in the presence of plate bound anti-CD3ε (0.5 mg/ml) and soluble anti-CD28 (0.5 mg/ml) for 4 days in different Tconv:Treg ratios to address the Treg suppressive capacities. (A) Representative histogram depicting a CFSE dilution assay to evaluate Tconv proliferation in presence of either WT or mPGES-1-deficient Treg cells (1:2 Tconv:Treg ratio) and Tconv alone. (B) Summary of proliferating percentages at different Tconv:Treg ratios. Shown is one of two representative experiments. [file Image_1.TIF]
